# Guidance to 2018 good practice: ARIA digitally-enabled, integrated, person-centred care for rhinitis and asthma

**DOI:** 10.1186/s13601-019-0252-0

**Published:** 2019-03-11

**Authors:** J. Bousquet, A. Bedbrook, W. Czarlewski, G. L. Onorato, S. Arnavielhe, D. Laune, E. Mathieu-Dupas, J. Fonseca, E. Costa, O. Lourenço, M. Morais-Almeida, A. Todo-Bom, M. Illario, E. Menditto, G. W. Canonica, L. Cecchi, R. Monti, L. Napoli, M. T. Ventura, G. De Feo, W. J. Fokkens, N. H. Chavannes, S. Reitsma, A. A. Cruz, J. da Silva, F. S. Serpa, D. Larenas-Linnemann, J. M. Fuentes Perez, Y. R. Huerta-Villalobos, D. Rivero-Yeverino, E. Rodriguez-Zagal, A. Valiulis, R. Dubakiene, R. Emuzyte, V. Kvedariene, I. Annesi-Maesano, H. Blain, P. Bonniaud, I. Bosse, Y. Dauvilliers, P. Devillier, J. F. Fontaine, J. L. Pépin, N. Pham-Thi, F. Portejoie, R. Picard, N. Roche, C. Rolland, P. Schmidt-Grendelmeier, P. Kuna, B. Samolinski, J. M. Anto, V. Cardona, J. Mullol, H. Pinnock, D. Ryan, A. Sheikh, S. Walker, S. Williams, S. Becker, L. Klimek, O. Pfaar, K. C. Bergmann, R. Mösges, T. Zuberbier, R. E. Roller-Wirnsberger, P. V. Tomazic, T. Haahtela, J. Salimäki, S. Toppila-Salmi, E. Valovirta, T. Vasankari, B. Gemicioğlu, A. Yorgancioglu, N. G. Papadopoulos, E. P. Prokopakis, I. G. Tsiligianni, S. Bosnic-Anticevich, R. O’Hehir, J. C. Ivancevich, H. Neffen, M. E. Zernotti, I. Kull, E. Melén, M. Wickman, C. Bachert, P. W. Hellings, G. Brusselle, S. Palkonen, C. Bindslev-Jensen, E. Eller, S. Waserman, L. P. Boulet, J. Bouchard, D. K. Chu, H. J. Schünemann, M. Sova, G. De Vries, M. van Eerd, I. Agache, I. J. Ansotegui, M. Bewick, T. Casale, M. Dykewick, M. Ebisawa, R. Murray, R. Naclerio, Y. Okamoto, D. V. Wallace, The MASK study group, J. Bousquet, P. W. Hellings, W. Aberer, I. Agache, C. A. Akdis, M. Akdis, M. R. Aliberti, R. Almeida, F. Amat, R. Angles, I. Annesi-Maesano, I. J. Ansotegui, J. M. Anto, S. Arnavielle, E. Asayag, A. Asarnoj, H. Arshad, F. Avolio, E. Bacci, C. Bachert, I. Baiardini, C. Barbara, M. Barbagallo, I. Baroni, B. A. Barreto, X. Basagana, E. D. Bateman, M. Bedolla-Barajas, A. Bedbrook, M. Bewick, B. Beghé, E. H. Bel, K. C. Bergmann, K. S. Bennoor, M. Benson, L. Bertorello, A. Z. Białoszewski, T. Bieber, S. Bialek, C. Bindslev-Jensen, L. Bjermer, H. Blain, F. Blasi, A. Blua, M. Bochenska Marciniak, I. Bogus-Buczynska, A. L. Boner, M. Bonini, S. Bonini, C. S. Bosnic-Anticevich, I. Bosse, J. Bouchard, L. P. Boulet, R. Bourret, P. J. Bousquet, F. Braido, V. Briedis, C. E. Brightling, J. Brozek, C. Bucca, R. Buhl, R. Buonaiuto, C. Panaitescu, M. T. Burguete Cabañas, E. Burte, A. Bush, F. Caballero-Fonseca, D. Caillaud, D. Caimmi, M. A. Calderon, P. A. M. Camargos, T. Camuzat, G. Canfora, G. W. Canonica, V. Cardona, K. H. Carlsen, P. Carreiro-Martins, A. M. Carriazo, W. Carr, C. Cartier, T. Casale, G. Castellano, L. Cecchi, A. M. Cepeda, N. H. Chavannes, Y. Chen, R. Chiron, T. Chivato, E. Chkhartishvili, A. G. Chuchalin, K. F. Chung, M. M. Ciaravolo, A. Ciceran, C. Cingi, G. Ciprandi, A. C. Carvalho Coehlo, L. Colas, E. Colgan, J. Coll, D. Conforti, J. Correia de Sousa, R. M. Cortés-Grimaldo, F. Corti, E. Costa, M. C. Costa-Dominguez, A. L. Courbis, L. Cox, M. Crescenzo, A. A. Cruz, A. Custovic, W. Czarlewski, S. E. Dahlen, G. D’Amato, C. Dario, J. da Silva, Y. Dauvilliers, U. Darsow, F. De Blay, G. De Carlo, T. Dedeu, M. de Fátima Emerson, G. De Feo, G. De Vries, B. De Martino, N. P. Motta Rubina, D. Deleanu, P. Demoly, J. A. Denburg, P. Devillier, S. Di Capua Ercolano, N. Di Carluccio, A. Didier, D. Dokic, M. G. Dominguez-Silva, H. Douagui, G. Dray, R. Dubakiene, S. R. Durham, G. Du Toit, M. S. Dykewicz, Y. El-Gamal, P. Eklund, E. Eller, R. Emuzyte, J. Farrell, A. Farsi, J. Ferreira de Mello, J. Ferrero, A. Fink-Wagner, A. Fiocchi, W. J. Fokkens, J. A. Fonseca, J. F. Fontaine, S. Forti, J. M. Fuentes-Perez, J. L. Gálvez-Romero, A. Gamkrelidze, J. Garcia-Aymerich, C. Y. García-Cobas, M. H. Garcia-Cruz, B. Gemicioğlu, S. Genova, G. Christoff, J. E. Gereda, R. Gerth van Wijk, R. M. Gomez, J. Gómez-Vera, S. González Diaz, M. Gotua, I. Grisle, M. Guidacci, N. A. Guldemond, Z. Gutter, M. A. Guzmán, T. Haahtela, J. Hajjam, L. Hernández, J. O’. B. Hourihane, Y. R. Huerta-Villalobos, M. Humbert, G. Iaccarino, M. Illario, Z. Ispayeva, J. C. Ivancevich, E. J. Jares, E. Jassem, S. L. Johnston, G. Joos, K. S. Jung, J. Just, M. Jutel, I. Kaidashev, O. Kalayci, A. F. Kalyoncu, J. Karjalainen, P. Kardas, T. Keil, P. K. Keith, M. Khaitov, N. Khaltaev, J. Kleine-Tebbe, L. Klimek, M. L. Kowalski, M. Kuitunen, I. Kull, P. Kuna, M. Kupczyk, V. Kvedariene, E. Krzych-Fałta, P. Lacwik, D. Larenas-Linnemann, D. Laune, D. Lauri, J. Lavrut, L. T. T. Le, M. Lessa, G. Levato, J. Li, P. Lieberman, A. Lipiec, B. Lipworth, K. C. Lodrup Carlsen, R. Louis, O. Lourenço, J. A. Luna-Pech, A. Magnan, B. Mahboub, D. Maier, A. Mair, I. Majer, J. Malva, E. Mandajieva, P. Manning, E. De Manuel Keenoy, G. D. Marshall, M. R. Masjedi, J. F. Maspero, E. Mathieu-Dupas, J. J. Matta Campos, A. L. Matos, M. Maurer, S. Mavale-Manuel, O. Mayora, M. A. Medina-Avalos, E. Melén, E. Melo-Gomes, E. O. Meltzer, E. Menditto, J. Mercier, N. Miculinic, F. Mihaltan, B. Milenkovic, G. Moda, M. D. Mogica-Martinez, Y. Mohammad, I. Momas, S. Montefort, R. Monti, D. Mora Bogado, M. Morais-Almeida, F. F. Morato-Castro, R. Mösges, A. Mota-Pinto, P. Moura Santo, J. Mullol, L. Münter, A. Muraro, R. Murray, R. Naclerio, R. Nadif, M. Nalin, L. Napoli, L. Namazova-Baranova, H. Neffen, V. Niedeberger, K. Nekam, A. Neou, A. Nieto, L. Nogueira-Silva, M. Nogues, E. Novellino, T. D. Nyembue, R. E. O’Hehir, C. Odzhakova, K. Ohta, Y. Okamoto, K. Okubo, G. L. Onorato, M. Ortega Cisneros, S. Ouedraogo, I. Pali-Schöll, S. Palkonen, P. Panzner, N. G. Papadopoulos, H. S. Park, A. Papi, G. Passalacqua, E. Paulino, R. Pawankar, S. Pedersen, J. L. Pépin, A. M. Pereira, M. Persico, O. Pfaar, J. Phillips, R. Picard, B. Pigearias, I. Pin, C. Pitsios, D. Plavec, W. Pohl, T. A. Popov, F. Portejoie, P. Potter, A. C. Pozzi, D. Price, E. P. Prokopakis, R. Puy, B. Pugin, R. E. Pulido Ross, M. Przemecka, K. F. Rabe, F. Raciborski, R. Rajabian-Soderlund, S. Reitsma, I. Ribeirinho, J. Rimmer, D. Rivero-Yeverino, J. A. Rizzo, M. C. Rizzo, C. Robalo-Cordeiro, F. Rodenas, X. Rodo, M. Rodriguez Gonzalez, L. Rodriguez-Mañas, C. Rolland, S. Rodrigues Valle, M. Roman Rodriguez, A. Romano, E. Rodriguez-Zagal, G. Rolla, R. E. Roller-Wirnsberger, M. Romano, J. Rosado-Pinto, N. Rosario, M. Rottem, D. Ryan, H. Sagara, J. Salimäki, B. Samolinski, M. Sanchez-Borges, J. Sastre-Dominguez, G. K. Scadding, H. J. Schunemann, N. Scichilone, P. Schmid-Grendelmeier, F. S. Serpa, S. Shamai, A. Sheikh, M. Sierra, F. E. R. Simons, V. Siroux, J. C. Sisul, I. Skrindo, D. Solé, D. Somekh, M. Sondermann, T. Sooronbaev, M. Sova, M. Sorensen, M. Sorlini, O. Spranger, C. Stellato, R. Stelmach, R. Stukas, J. Sunyer, J. Strozek, A. Szylling, J. N. Tebyriçá, M. Thibaudon, T. To, A. Todo-Bom, P. V. Tomazic, S. Toppila-Salmi, U. Trama, M. Triggiani, C. Suppli Ulrik, M. Urrutia-Pereira, R. Valenta, A. Valero, A. Valiulis, E. Valovirta, M. van Eerd, E. van Ganse, M. van Hague, O. Vandenplas, M. T. Ventura, G. Vezzani, T. Vasankari, A. Vatrella, M. T. Verissimo, F. Viart, G. Viegi, D. Vicheva, T. Vontetsianos, M. Wagenmann, S. Walker, D. Wallace, D. Y. Wang, S. Waserman, T. Werfel, M. Westman, M. Wickman, D. M. Williams, S. Williams, N. Wilson, J. Wright, P. Wroczynski, P. Yakovliev, B. P. Yawn, P. K. Yiallouros, A. Yorgancioglu, O. M. Yusuf, H. J. Zar, L. Zhang, N. Zhong, M. E. Zernotti, I. Zhanat, M. Zidarn, T. Zuberbier, C. Zubrinich, A. Zurkuhlen

**Affiliations:** 1 MACVIA-France, Fondation Partenariale FMC VIA-LR, CHU Arnaud de Villeneuve, 371 Avenue du Doyen Gaston Giraud, 34295 Montpellier Cedex 5, France; 2 INSERM U 1168, VIMA: Ageing and Chronic Diseases Epidemiological and Public Health Approaches, Villejuif, Université Versailles St-Quentin-en-Yvelines, UMR-S 1168, Montigny Le Bretonneux, France; 3 Euforea, Brussels, Belgium; 4 Humboldt-Universität zu Berlin, Berlin Institute of Health, Comprehensive Allergy Center, Department of Dermatology and Allergy, Charité, Universitätsmedizin Berlin, Berlin, Germany; 5 Medical Consulting Czarlewski, Levallois, France; 6 KYomed INNOV, Montpellier, France; 7 Center for Research in Health Technology and Information Systems, Faculdade de Medicina da Universidade do Porto, Medida, Lda Porto, Portugal; 8 UCIBIO, REQUINTE, Faculty of Pharmacy and Competence Center on Active and Healthy Ageing, University of Porto (Porto4Ageing), Porto, Portugal; 9 Faculty of Health Sciences and CICS – UBI, Health Sciences Research Centre, University of Beira Interior, Covilhã, Portugal; 10 Allergy Center, CUF Descobertas Hospital, Lisbon, Portugal; 11 Imunoalergologia, Centro Hospitalar Universitário de Coimbra and Faculty of Medicine, University of Coimbra, Coimbra, Portugal; 12 Division for Health Innovation, Campania Region and Federico II University Hospital Naples (R&D and DISMET), Naples, Italy; 13 CIRFF, Federico II University, Naples, Italy; 14 Personalized Medicine Clinic Asthma and Allergy, Humanitas Research Hospital, Humanitas University, Rozzano, Milan, Italy; 15 SOS Allergology and Clinical Immunology, USL Toscana Centro, Prato, Italy; 16 Department of Medical Sciences, Allergy and Clinical Immunology Unit, University of Torino & Mauriziano Hospital, Turin, Italy; 17 Consortium of Pharmacies and Services COSAFER, Salerno, Italy; 18 Unit of Geriatric Immunoallergology, University of Bari Medical School, Bari, Italy; 19 Department of Medicine, Surgery and Dentistry “Scuola Medica Salernitana”, University of Salerno, Salerno, Italy; 20 Department of Otorhinolaryngology, Amsterdam University Medical Centre (AMC), Amsterdam, The Netherlands; 21 Department of Public Health and Primary Care, Leiden University Medical Center, Leiden, The Netherlands; 22 ProAR – Nucleo de Excelencia em Asma, Federal University of Bahia, Vitória da Conquista, Brazil; 23 WHO GARD Planning Group, Salvador, Brazil; 24 Department of Internal Medicine and Allergic Clinic of Professor Polydoro Ernani de Sao, Thiago University Hospital, Federal University of Santa Catarina (UFSC), Florianópolis, Brazil; 25 Asthma Reference Center, Escola Superior de Ciencias da Santa Casa de Misericordia de Vitoria, Vitória, Esperito Santo Brazil; 26 Center of Excellence in Asthma and Allergy, Médica Sur Clinical Foundation and Hospital, Mexico City, Mexico; 27 Hospital General Regional 1 “Dr Carlos Mc Gregor Sanchez Navarro” IMSS, Mexico City, Mexico; 28 Allergist, Mexico City, Mexico; 29 Clinic of Children’s Diseases, and Institute of Health Sciences Department of Public Health, Vilnius University Institute of Clinical Medicine, Vilnius, Lithuania; 30 European Academy of Paediatrics (EAP/UEMS-SP), Brussels, Belgium; 31 Clinic of Infectious, Chest Diseases, Dermatology and Allergology, Vilnius University, Vilnius, Lithuania; 32 Clinic of Children’s Diseases, Faculty of Medicine, Vilnius University, Vilnius, Lithuania; 33 Faculty of Medicine, Vilnius University, Vilnius, Lithuania; 34 Epidemiology of Allergic and Respiratory Diseases, Department Institute Pierre Louis of Epidemiology and Public Health, INSERM, Sorbonne Université, Medical School Saint Antoine, Paris, France; 35 Department of Geriatrics, Montpellier University Hospital, Montpellier, France; 36 EA 2991, Euromov, University Montpellier, Montpellier, France; 37 CHU Dijon, Dijon, France; 38 Allergist, La Rochelle, France; 39 Sleep Unit, Department of Neurology, Hôpital Gui-de-Chauliac Montpellier, Montpellier, France; 40 Inserm U1061, Montpellier, France; 41 UPRES EA220, Pôle des Maladies des Voies Respiratoires, Hôpital Foch, Université Paris-Saclay, Suresnes, France; 42 Allergist, Reims, France; 43 Laboratoire HP2, Grenoble, INSERM, U1042, Université Grenoble Alpes, Grenoble, France; 44 CHU de Grenoble, Grenoble, France; 45 Allergy Department, Pasteur Institute, Paris, France; 46 Conseil Général de l’Economie Ministère de l’Economie, de l’Industrie et du Numérique, Paris, France; 47 Pneumologie et Soins Intensifs Respiratoires, Hôpitaux Universitaires Paris, Centre Hôpital Cochin, Paris, France; 48 Association Asthme et Allergie, Paris, France; 49 Allergy Unit, Department of Dermatology, University Hospital of Zurich, Zurich, Switzerland; 50 Division of Internal Medicine, Asthma and Allergy, Barlicki University Hospital, Medical University of Lodz, Lodz, Poland; 51 Department of Prevention of Envinronmental Hazards and Allergology, Medical University of Warsaw, Warsaw, Poland; 52 ISGlobAL, Centre for Research in Environmental Epidemiology (CREAL), Barcelona, Spain; 53 IMIM (Hospital del Mar Research Institute), Barcelona, Spain; 54 CIBER Epidemiología y Salud Pública (CIBERESP), Barcelona, Spain; 55 Universitat Pompeu Fabra (UPF), Barcelona, Spain; 56 Allergy Section, Department of Internal Medicine, Hospital Vall ‘dHebron & ARADyAL Research Network, Barcelona, Spain; 57 Rhinology Unit and Smell Clinic, ENT Department, Hospital Clínic, University of Barcelona, Barcelona, Spain; 58 Clinical and Experimental Respiratory Immunoallergy, IDIBAPS, CIBERES, University of Barcelona, Barcelona, Spain; 59 Asthma UK Centre for Applied Research, The Usher Institute of Population Health Sciences and Informatics, The University of Edinburgh, Edinburgh, UK; 60 Honorary Clinical Research Fellow, Allergy and Respiratory Research Group, Usher Institute of Population Health Sciences and Informatics, Medical School, University of Edinburgh, Edinburgh, UK; 61 The Usher Institute of Population Health Sciences and Informatics, The University of Edinburgh, Edinburgh, UK; 62 Asthma UK, Mansell Street, London, UK; 63 International Primary Care Respiratory Group IPCRG, Aberdeen, Scotland, UK; 64 Department of Otolaryngology, Head and Neck Surgery, University of Mainz, Mainz, Germany; 65 Center for Rhinology and Allergology, Wiesbaden, Germany; 66 Department of Otorhinolaryngology, Head and Neck Surgery, Section of Rhinology and Allergy, University Hospital Marburg, Phillipps-Universität Marburg, Marburg, Germany; 67 Corporate Member of Freie Universität Berlin, Humboldt-Uniersität zu Berlin, Charité - Universitätsmedizin Berlin, Berlin, Germany; 68 Berlin Institute of Health, Comprehensive Allergy-Centre, Department of Dermatology and Allergy, Member of GA2LEN, Berlin, Germany; 69 Institute of Medical Statistics, and Computational Biology, Medical Faculty, University of Cologne, Cologne, Germany; 70 CRI-Clinical Research International-Ltd, Hamburg, Germany; 71 Department of Internal Medicine, Medical University of Graz, Graz, Austria; 72 Department of ENT, Medical University of Graz, Graz, Austria; 73 Skin and Allergy Hospital, Helsinki University Hospital, University of Helsinki, Helsinki, Finland; 74 Association of Finnish Pharmacies, Helsinki, Finland; 75 Department of Lung Diseases and Clinical Immunology, Terveystalo Allergy Clinic, University of Turku, Turku, Finland; 76 FILHA, Finnish Lung Association, Helsinki, Finland; 77 Department of Pulmonary Diseases, Cerrahpasa Faculty of Medicine, Istanbul University-Cerrahpasa, Istambul, Turkey; 78 Department of Pulmonary Diseases, Faculty of Medicine, Celal Bayar University, Manisa, Turkey; 79 Division of Infection, Immunity and Respiratory Medicine, Royal Manchester Children’s Hospital, University of Manchester, Manchester, UK; 80 Allergy Department, 2nd Pediatric Clinic, Athens General Children’s Hospital “P&A Kyriakou”, University of Athens, Athens, Greece; 81 Department of Otorhinolaryngology, University of Crete School of Medicine, Heraklion, Greece; 82 Health Planning Unit, Department of Social Medicine, Faculty of Medicine, University of Crete, Crete, Greece; 83 University of Sydney and Woolcock Emphysema Centre and Local Health District, Woolcock Institute of Medical Research, Glebe, NSW Australia; 84 Department of Allergy, Immunology and Respiratory Medicine, Alfred Hospital and Central Clinical School, Monash University, Melbourne, VIC Australia; 85 Department of Immunology, Monash University, Melbourne, VIC Australia; 86 Servicio de Alergia e Immunologia, Clinica Santa Isabel, Buenos Aires, Argentina; 87 Director of Center of Allergy, Immunology and Respiratory Diseases, Santa Fe, Argentina Center for Allergy and Immunology, Santa Fe, Argentina; 88 Universidad Católica de Córdoba, Córdoba, Argentina; 89 Department of Clinical Science and Education, Södersjukhuset, Karolinska Institutet, Stockholm, Sweden; 90 Sachs’ Children and Youth Hospital, Södersjukhuset, Stockholm and Institute of Environmental Medicine, Karolinska Institutet, Stockholm, Sweden; 91 Centre for Clinical Research Sörmland, Uppsala University, Eskilstuna, Sweden; 92 Upper Airways Research Laboratory, ENT Dept, Ghent University Hospital, Ghent, Belgium; 93 Department of Otorhinolaryngology, Univ Hospitals Leuven, Louvain, Belgium; 94 Academic Medical Center, Univ of Amsterdam, Amsterdam, The Netherlands; 95 Department of Respiratory Medicine, Ghent University Hospital, Ghent, Belgium; 96 EFA European Federation of Allergy and Airways Diseases Patients’ Associations, Brussels, Belgium; 97 Department of Dermatology and Allergy Centre, Odense University Hospital, Odense Research Center for Anaphylaxis (ORCA), Odense, Denmark; 98 Department of Medicine, Clinical Immunology and Allergy, McMaster University, Hamilton, ON Canada; 99 Quebec Heart and Lung Institute, Laval University, Québec City, QC Canada; 100 Clinical Medecine, Laval’s University, Quebec City, Canada; 101 Medecine Department, Hôpital de la Malbaie, Quebec, Canada; 102 Department of Health Research Methods, Evidence and Impact, Division of Immunology and Allergy, McMaster University, Hamilton, ON Canada; 103 Department of Respiratory Medicine, University Hospital Olomouc, Olomouc, Czech Republic; 104 Peercode BV, Geldermalsen, The Netherlands; 105 Faculty of Medicine, Transylvania University, Brasov, Romania; 106 Department of Allergy and Immunology, Hospital Quirón Bizkaia, Erandio, Spain; 107 iQ4U Consultants Ltd, London, UK; 108 Division of Allergy/Immunology, University of South Florida, Tampa, USA; 109 Section of Allergy and Immunology, Saint Louis University School of Medicine, Saint Louis, MO USA; 110 Clinical Reserch Center for Allergy and Rheumatology, Sagamihara National Hospital, Sagamihara, Japan; 111 Medical Communications Consultant, MedScript Ltd (Ireland & New Zealand), Dundalk, Ireland; 112 Honorary Research Fellow, OPC, Cambridge, UK; 113 Johns Hopkins School of Medicine, Baltimore, MD USA; 114 Department of Otorhinolaryngology, Chiba University Hospital, Chiba, Japan; 115 Nova Southeastern University, Fort Lauderdale, FL USA

**Keywords:** App, Asthma, Care pathways, MASK, mHealth, Rhinitis, DG Santé

## Abstract

**Aims:**

Mobile Airways Sentinel NetworK (MASK) belongs to the Fondation Partenariale MACVIA-LR of Montpellier, France and aims to provide an active and healthy life to rhinitis sufferers and to those with asthma multimorbidity across the life cycle, whatever their gender or socio-economic status, in order to reduce health and social inequities incurred by the disease and to improve the digital transformation of health and care. The ultimate goal is to change the management strategy in chronic diseases.

**Methods:**

MASK implements ICT technologies for individualized and predictive medicine to develop novel care pathways by a multi-disciplinary group centred around the patients.

**Stakeholders:**

Include patients, health care professionals (pharmacists and physicians), authorities, patient’s associations, private and public sectors.

**Results:**

MASK is deployed in 23 countries and 17 languages. 26,000 users have registered.

**EU grants (2018):**

MASK is participating in EU projects (POLLAR: impact of air POLLution in Asthma and Rhinitis, EIT Health, DigitalHealthEurope, Euriphi and Vigour).

**Lessons learnt:**

(i) Adherence to treatment is the major problem of allergic disease, (ii) Self-management strategies should be considerably expanded (behavioural), (iii) Change management is essential in allergic diseases, (iv) Education strategies should be reconsidered using a patient-centred approach and (v) Lessons learnt for allergic diseases can be expanded to chronic diseases.

## Introduction

In all societies, the burden and cost of allergic and chronic respiratory diseases (CRDs) is increasing rapidly. Most economies are struggling to deliver modern health care effectively. There is a need to support the transformation of the health care system for integrated care with organizational health literacy. MASK (Mobile Airways Sentinel Network) [1] is a new development of the ARIA (Allergic Rhinitis and its Impact on Asthma) initiative [2, 3]. It works closely with POLLAR (Impact of Air POLLution on Asthma and Rhinitis, EIT Health) [4], and collaborates with professional and patient organizations in the field of allergy and airway diseases. MASK proposes real-life care pathways (ICPs) centred around the patient with rhinitis and/or asthma multimorbidity. It uses mHealth monitoring of environmental exposure and considers biodiversity. With the help of three EU projects (DigitalHealthEurope, Eurifi and Vigour) recently accepted on the digital transformation of health, MASK proposes a second change management strategy. The first one was the ARIA change management associated with the recognition and wide acceptance by all stakeholders of the essential links between rhinitis and asthma. The second one deals with change management of care pathways for rhinitis and asthma [5].

In the context of implementing communication on the digital transformation of health and care, specifically in relation to chapter 5 of the document “Digital tools for citizen empowerment and for person-centred care”, DG SANTE has taken steps towards supporting the scaling-up and wider implementation of good practices in the field of digitally-enabled, integrated, person-centred care. This work was carried out in collaboration with the newly-established Commission Expert Group, the “Steering Group on Health Promotion, Disease Prevention and Management of Non-Communicable Diseases”.

For this purpose, DG SANTE—in collaboration with the Commission’s Joint Research Centre—organized a “marketplace” workshop with the Joint Research Centre in Ispra, the third biggest European Commission site after Brussels and Luxembourg. The aim of this workshop was for representatives from Member States and other countries participating in the 3rd Health Programme to learn more about the 10 good practices and key policy initiatives in the domain of digitally-enabled, integrated, person-centred care, with a view to possible transfer and replication of the presented practices.

The current paper reviews the questions raised during the workshop concerning the good practice on allergic rhinitis and asthma: ARIA digitally-enabled, integrated, person-centred care for rhinitis and asthma multimorbidity using real-world evidence [1]. This practice is a GARD (Global Alliance against Chronic Respiratory Diseases) demonstration project.

### The practice

**Fig. 1:**
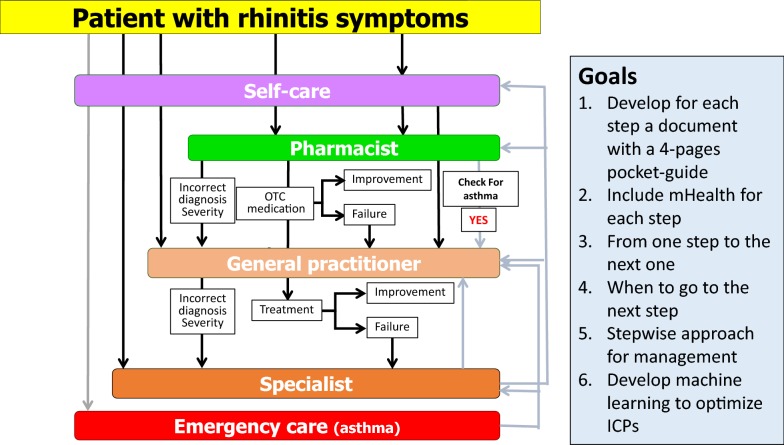
Care pathways for chronic respiratory diseases. From [6–8]

**Fig. 2:**
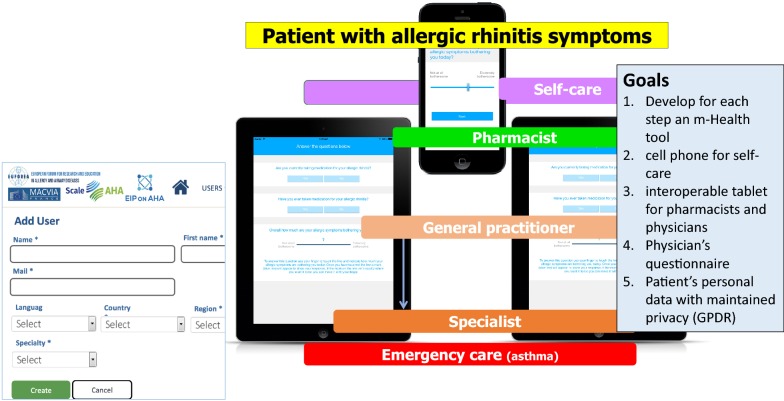
ICT solutions embedded in care pathways for chronic respiratory diseases

The practice includes the care pathways defined in 2014 [6–8] (Fig. 1) as well as ICT (Information and Communication Technology) solutions (cell phones for patients, inter-operable tablets for health care professionals and a web-based questionnaire for physicians) [1, 9] (Fig. 2). The aim is to develop a change management strategy for chronic diseases [5].

MASK is a patient-centred ICT system [8]. A mobile phone app (the *Allergy Diary*, now called MASK-air), central to MASK, is available in 23 countries. It has been validated [10] and found to be an easy and effective method of assessing the symptoms of allergic rhinitis (AR) and work productivity [10–13]. MASK follows the checklist for the evaluation of Good Practices developed by the European Union Joint Action JA-CHRODIS (Joint Action on Chronic Diseases and Promoting Healthy Ageing across the Life Cycle) [14]. One of the major aims of MASK is to provide care pathways [15] in rhinitis and asthma multimorbidity [16] including a sentinel network using the geolocation of users [17]. It can also inform the App users of the pollen and/or pollution risk level in their area, by means of geolocation (Table 1).

**Table 1:** The ICT solution

App (MASK-air) deployed in 23 countries: TRL9 (Technology Readiness level), Electronic clinical decision support system (ARIA e-CDSS): TRL 7, e-physician questionnaire deployed in 16 countries: TRL9
MASK-air good practice [1, 14]
5-year work
App: 26,000 users, 23 countries, 17 languages
GDPR including geolocation [105]
GP of the EIP on AHA, follows CHRODIS [14]
Based on 11 EU grants (MeDALL [106], GA^2^LEN [107]) including—in 2018—POLLAR [4], VIGOUR, DigitalHealthEurope and Euriphi
From a validated “research” tool (2004-2018) to large scale deployment (2019–)
Validation with COSMIN guidelines [40]
Baseline characteristics [12]
Work productivity [41, 42]
EQ-5D [43]
Novel phenotypes of allergic diseases [44]
Adherence to treatment and novel approaches to inform the efficacy of treatment [45].
Patient’s organizations and scientific societies involved
GARD (WHO alliance)
Presented during WHO and EU ministerial meetings
Next-generation care pathways meeting (Dec 3, 2018) with the EIP on AHA, POLLAR (EIT Health) and GARD
47 MASK papers in 12 languages [99, 108, 109]
Dissemination according to the EIP on AHA [26]
Transfer of innovation (TWINNING [110])
Interoperable platform with MASK
25 RS plus Argentina, Australia, Brazil, Canada, Mexico [99, 108, 109]
700 patients enrolled
GDPR solutions being solved
ARIA e-CDSS [9, 111]
Interoperable platform with MASK
Based on an expert meeting
Electronic version available
GDPR solutions being solved
Developments
App for home services
App for sleep
App for COPD
App for other chronic diseases

The practice has been developed for allergic rhinitis (and asthma multimorbidity), being the most common chronic disease globally [18, 19] and affecting all age groups from early childhood to old age. There are several unmet needs that should be addressed in an ICP. Moreover, the lessons learnt will benefit all chronic diseases since rhinitis is considered as a mild disease although it impairs social life, school and work productivity considerably [20]. It is estimated that, in the EU, work loss accounts for 30–100 b€ annually. Moreover, it is essential to consider mild chronic diseases and to establish health promotion and management strategies early in life in order to prevent a severe outcome and to promote healthy ageing [21].

#### Level of care integration

MASK is used for the integration of primary and specialist care, of primary-secondary-tertiary health care, as well as of health and social care for disease management.

#### Deployment

Many of the GPs that are developed in one region (country) take into account health systems, availability of treatments and legal considerations which makes it difficult to scale up the practice without customization. MASK has taken the opposite direction starting with a tool immediately available in 10 languages and 14 countries and regularly scaled up. Moreover, the tool is included in a generic ICP (Fig. 2) that can be customized easily in any country globally.

### Geographical scope of the practice

MASK was developed in English and is currently available in 23 countries and 17 languages (Table 2).

**Table 2:**
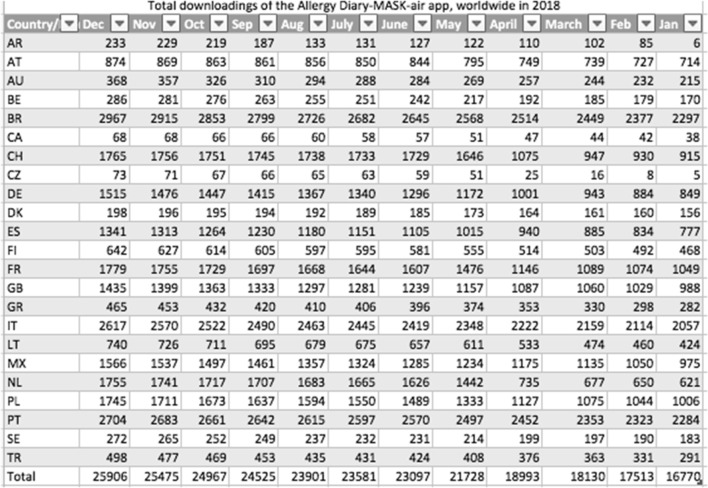
List of countries using MASK-air

*AR* Argentina, *AT* Austria, *AU* Australia, *Be* Belgium, *BR* Brazil, *CA* Canada, *CH* Switzerland, *CZ* Czech Republic, *DE* Germany, *DK* Denmark, *ES* Spain, *FI* Finland, *FR* France, *GB* Great Britain, *GR* Greece, *IT* Italy, *LT* Lithuania, *MX* Mexico, *NL* The Netherlands, *PL* Poland, *PT* Portugal, *SE* Sweden, *TR* Turkey

#### New countries

Deployment is in process in Bolivia, Colombia, Japan and Peru. The involvement of developing countries is needed to offer a practice for middle- and low-income countries that will benefit poverty areas of developed countries and that will be in line with the mission of GARD. Deployment to the US is being discussed with the National Institute for Allergy and Infectious diseases (NIH).

#### Transfer of innovation of allergic rhinitis and asthma multimorbidity in the elderly (MASK Reference Site Twinning, EIP on AHA)

The EIP on AHA includes 74 Reference Sites. The aim of this TWINNING is to transfer innovation from the MASK App to other reference sites. The phenotypic characteristics of rhinitis and asthma multimorbidity in adults and the elderly have been compared using validated mHealth tools (i.e. the Allergy Diary and CARAT [22]) in 23 Reference Sites or regions across Europe, Argentina, Australia, Brazil and Mexico [23].

### Individuals/institutions reached

ARIA has been implemented in over 70 countries globally [3], and several governments use the practice. Approximately 26,000 users have registered to the MASK database. 700 patients have been enrolled in the Twinning. Due to privacy, there is no possibility of assessing users who have reported data.

### Timeframe

The project was initiated in 1999 during a World Health Organization (WHO) workshop (ARIA) and undergoes continuous developments. The ARIA initiative, commenced during a WHO workshop in 1999 [2], has been further developed by the WHO Collaborating Center for Asthma and Rhinitis (2002–2013). The initial goals (Phase 1) were (1) to propose a new AR classification, (2) to promote the concept of multimorbidity in asthma and rhinitis and (3) to develop guidelines with all stakeholders that could be used globally for all countries and all populations. ARIA has been disseminated and implemented in over 70 countries [3, 19, 24–32]. It was developed as a guideline [19] using the GRADE approach [33–39].

MASK, the Phase 3 ARIA initiative, is focusing on (1) the implementation of multi-sectoral care pathways (2) using emerging technologies (3) with real world data (4) for individualized and predictive medicine (5) in rhinitis and asthma multimorbidity (6) by a multi-disciplinary group or by patients themselves (self-care) using the AIRWAYS ICPs algorithm (7) across the life cycle [8, 17]. It will be scaled up using the EU EIP on AHA strategy [26].

Phase 4 began in 2018. It concerns “change management” and includes the impact of air pollution in asthma and rhinitis (EIT Health 2018–2019: POLLAR, Impact of Air POLLution in Asthma and Rhinitis) [4] as well as the digital transformation of health and care (DigitalHealthEurope, Euriphi and Vigour).

Developments for 2019 include a multimorbidity App and the deployment of an app for home services.

The MASK project is intended to be sustainable and a business plan has been initiated.

The medium-term future is to develop care pathways for the prevention and control of chronic diseases to sustain planetary health. A symposium during the Finnish Presidency of the EU Council is planned for October 2019.

### Scientific evidence and conceptual framework for configuring the practice

The scientific evidence is based on a validated “research” tool (The Allergy Diary, –2018) that has led to large scale deployment (MASK-air, 2019–):

- Validation of the app using COSMIN guidelines [40].
- Baseline characteristics informed [12].
- Work productivity associated with the control of allergic diseases [41, 42].
- EQ-5D is available and has been found to correlate to baseline characteristics [43].
- Novel phenotypes of allergic diseases have been discovered [44].
- Adherence to treatment is extremely low and novel approaches to inform the efficacy of treatment have been proposed [45] leading to novel studies for a better understanding of guidelines [46, 47].

### Evidence of impact

MASK has identified novel phenotypes of allergic diseases [44] that have been confirmed in classical epidemiologic studies by re-analyzing them [48–51]. One of the studies used the MASK baseline characteristics [49]. These phenotypes allowed the re-classification of allergic multimorbidity and the discovery of a new extreme phenotype of allergic diseases that need to be considered in the stratification of patients.

MASK has shown real-life mHealth data for the first time in allergy treatment in 9,950 users [1, 45]. This led to next-generation care pathways for allergic diseases (meeting co-organized by POLLAR, a member of EIT Health, EIP on AHA and GARD (WHO alliance): 3-12-2018) and proposed a change management strategy [5].

MASK is involved in an EIT Health project (POLLAR) which assesses the interactions between air pollution, asthma and rhinitis [4].

With the EIP on AHA, MASK is involved in 3 EU projects on the digital transformation of health and care (DigiHealthEurope, Euriphi and Vigour).

MASK is also involved in a large project on Planetary Health in a side event which will take place during the Presidency of the EU council (Finland). This event will gather researchers, academic leaders and other experts from European institutions as well as other stakeholders and will discuss Planetary Health global challenges and their scientific solutions. Experts on human health as well as on effects of climate change, urbanization and food production will be invited to prepare a European initiative to promote effective and sustainable research on planetary health issues. The event similarly aims at raising political awareness about the need for multidisciplinary and systemic approaches to Planetary Health issues globally and in the EU. The multimorbid App developed by MASK may be used in the project.

### Contextual relevance

#### The practice addresses a public health priority

Chronic respiratory diseases (CRDs) are major non-communicable diseases (NCDs) [18]. Rhinitis and asthma multimorbidity is common and the two diseases should be considered jointly [19]. Asthma is the most common NCD in children and rhinitis is the most common chronic disease in Europe. They often start early in life, persist across the life cycle and cause a high disease burden in all age groups [19]. By 2020, rhinitis will affect at least 20% of the old age population [52–56]. These diseases represent an enormous burden associated to medical and social costs and they impact health and social inequalities.

#### The practice is based on a local/regional/national strategic action plan

The Polish Presidency of the EU Council (3051st Council Conclusions) made the prevention, early diagnosis and treatment of asthma and allergic diseases a priority to reduce health inequalities [57, 58]. The 3206th Cyprus Council Conclusions [59] recommended that the diagnosis and treatment of chronic diseases should be initiated as early as possible to improve AHA. Debates at the European Parliament recommended the early diagnosis and management of CRDs in order to promote active and healthy ageing (AHA) [60–62].

The practice is also a WHO-associated project: Initial workshop (1999), WHO Collaborating Center for rhinitis and asthma (2004–2014), Global Alliance against Chronic Respiratory Diseases (GARD) [63, 64] demonstration project (2015–).

### Unmet needs

Several unmet needs have been identified in allergic diseases. They include (1) suboptimal rhinitis and asthma control due to medical, cultural and social barriers [65, 66], (2) better understanding of endotypes [67], phenotypes and multimorbidities, (3) assessment of allergen and pollutants as risk factors to promote sentinel networks in care pathways, (4) stratification of patients for optimized care pathways [68] and (5) promotion of multidisciplinary teams within integrated care pathways, endorsing innovation in clinical trials and encouraging patient empowerment [17, 69].

### Overall goal

The general objective of AIRWAYS-ICPs [6–8] is to develop multi-sectoral ICPs for CRDs used across European countries and regions in order to (1) reduce the burden of the diseases in a patient-centred approach, (2) promote AHA, (3) create a care pathways simulator tool which can be applied across the life cycle and in older adults, (4) reduce health and social inequalities, (5) reduce gender inequalities, (6) use the lessons learned in CRDs for chronic diseases and (7) promote SDG3 (more specifically 3.4) (https://www.who.int/sdg/targets/en/). In September 2015, the UN General Assembly established the Sustainable Development Goals (SDGs), a set of global goals for fair and sustainable health at every level from planetary biosphere to local community [70, 71], essential for sustainable development. SDG 3 prioritizes health and well-being for all ages.

The aim of AIRWAYS-ICPs is also to generalise the approach of the uniform definition of severity, control and risk of severe asthma presented to WHO [66] and allergic diseases [72] in order to develop a uniform risk stratification usable for chronic diseases in most situations.

MASK further refined AIRWAYS ICPs using mobile technology to promote the digital transformation of health and care in developed and developing countries for all age groups.

### Target population

In the initial phase, the target population included all patients with allergic rhinitis and asthma multimorbidity. Rhinitis and asthma are considered as a model for all chronic diseases and the project is being extended to chronic diseases.

All patients able to use a smartphone (≥ 12 years) represent the target population. A special effort is being placed in underserved populations from developing countries as the practice is a GARD (Global Alliance against Chronic Respiratory Diseases, WHO alliance) demonstration project.

### Stakeholders involved

#### Involvement in the design, implementation (including the creation of ownership), evaluation, continuity/sustainability

As from the very first workshop in 1999, the ARIA initiative has included all stakeholders required to develop a WHO programme on CRDs (GARD). In particular, patient’s organizations were involved. All health care professionals were also involved (physicians, primary care, pharmacists, other health care professionals). Another important component of ARIA was the deployment to developing countries [73]. Moreover, policy makers were also actively involved.

ARIA has grown regularly over the past 20 years and an ARIA chapter is ongoing in over 70 countries in all continents with a very active scaling up strategy [26]. MASK has used the ARIA working group to scale up the practice.

#### All stakeholders were highly receptive

The ARIA and now the MASK community is very cohesive and all members are extremely reactive. They have been particularly active in deploying MASK in the 23 countries and we have received requests from many other countries in which MASK-air is not yet available.

#### Resistance or conflict of interest: None

### Implementation methodology/strategy

We used the scaling up strategy of the European Innovation Partnership on Active and Healthy Ageing and proposed a 5‐step framework for developing an individual: (1) what to scale up: (1‐a) databases of good practices, (1‐b) assessment of viability of the scaling up of good practices, (1‐c) classification of good practices for local replication and (2) how to scale up: (2‐a) facilitating partnerships for scaling up, (2‐b) implementation of key success factors and lessons learnt, including emerging technologies for individualized and predictive medicine. This strategy has already been applied to the chronic respiratory disease action plan of the European Innovation Partnership on Active and Healthy Ageing [26].

### Consistency in the pace of delivery

For the past 20 years, ARIA has been a success story in over 72 countries [3, 8, 19, 24, 25, 27, 28, 30–32, 38, 46, 74–100]. A Pocket Guide has been translated into 52 languages. MASK is following ARIA with the same group and the same strategy.

### Main outcomes and evaluation of the practice

**Fig. 3:**
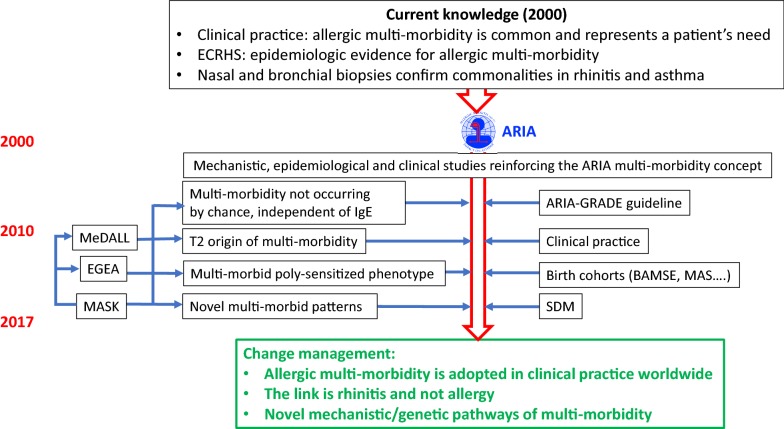
Change management strategy in ARIA Phases 1 and 2. From [5]

The ARIA strategy was to change management in the treatment of asthma and rhinitis since nasal symptoms—often the most troublesome—were not considered in most asthmatics. Over 85% of asthma in children and adolescents is associated with rhinitis, suggesting common pathways, whereas only 20–30% of rhinitis patients have asthma, suggesting rhinitis-specific genes. There is a link between asthma severity and rhinitis multimorbidity. Asthma is more severe in patients with rhinitis [101]. The strategy at all levels of care indicates that it is essential to consider multimorbidity in the management of asthma for the benefit of the patient and the satisfaction of the treatment as shown in many surveys (Fig. 3). Some studies have found that the ARIA strategy is more effective than free treatment choice [102]. Moreover, EMA has used the ARIA recommendations for the approval of a house dust mite immunotherapy tablet including asthma and rhinitis multimorbidity [103].

**Fig. 4:**
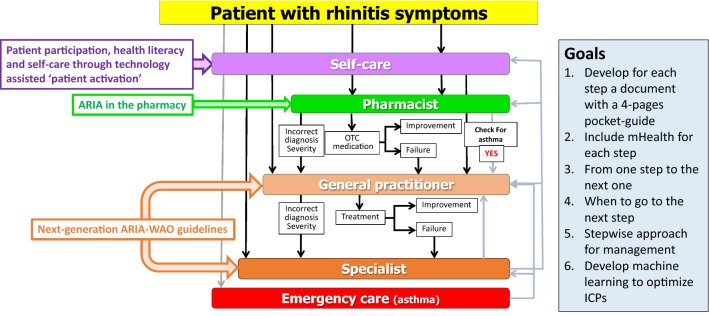
Next-generation care pathways. From [5]

**Fig. 5:**
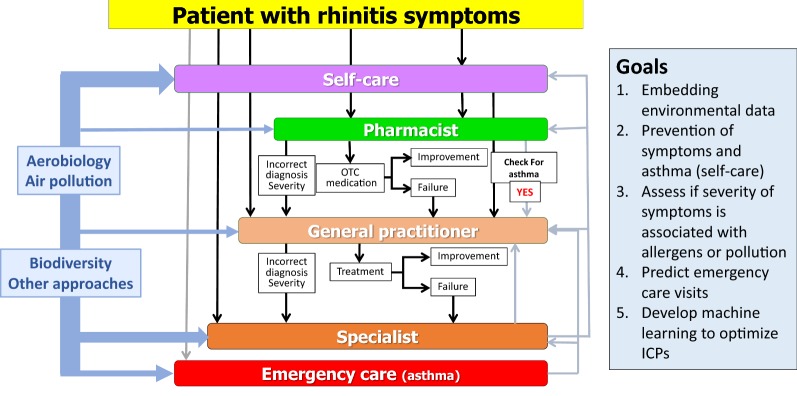
Embedding air pollution and biodiversity in care pathways. From [4]

The change management strategy of MASK has not yet been evaluated. However, the results of the first studies indicate that the vast majority of patients are not adherent to treatment [45] and that next-generation care pathways are needed (Figs. 4 and 5). Next-generation care pathways were initiated in Paris, December 3, 2018, as part of POLLAR, MASK and GARD.

#### Additional (secondary) outcomes assessed

Work productivity and school performance are measured. When rhinitis and/or asthma are not well controlled, work productivity is impaired [1, 41, 43].

### Sustainability of the practice

**Fig. 6:**
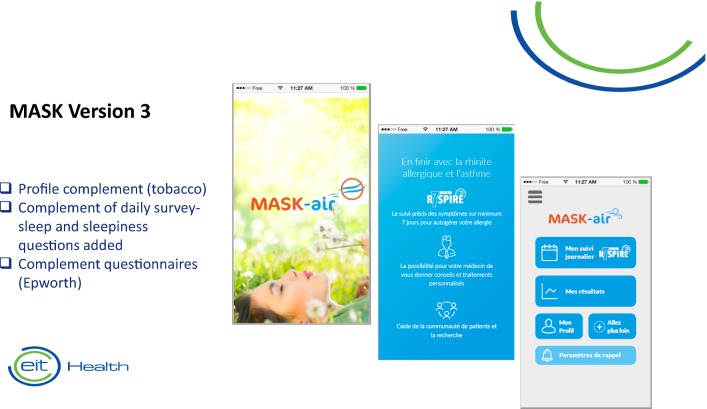
From *The Allergy Diary* to MASK-air

The MASK App, The *Allergy Diary*, was used to demonstrate the scientific value of the project [1]. It has been replaced by the commercial App, MASK-air, which is version 3.0 and which includes questionnaires (e.g. tobacco and allergens) and sleep (VAS and Epworth questionnaire [104]) (Fig. 6). A business plan is in place for the sustainability of the practice.

### Communication about the practice and dissemination of results

A communication strategy has been set up [1] and includes a website (mask-air.com), media coverage, leaflets and newsletters, publications in scientific journals and lay press, partners’ networks and events. The MASK community includes over 300 members in all countries in which MASK is deployed.

### Budget required to implement the practice

The budget required to implement the MASK strategy is around 1.5 M€. It will be provided by the private sector (1 M€) and from EU grants, in particular a Structural and Development Fund. POLLAR has an additive budget of 2 M€ to embed outdoor air pollution and aerobiology data in the ICP using artificial intelligence.

It is difficult to estimate human resources since many physicians worked in the 23 countries for the translation, adaptation of the practice and its implementation. It can be proposed that 50–100 h have been spent working in each country.

The practice has been presented to multiple national and international meetings.

Sustainability has been carefully evaluated and a business plan is in place.

### Main lessons learned

- Adherence to treatment is the major problem of allergic disease.
- Self-management strategies should be considerably expanded (behavioural).
- Change management is essential in allergic diseases.
- Education strategies should be reconsidered using a patient-centred approach.
- Lessons learned for allergic diseases can be expanded to chronic diseases.

### Improvement and expansion of the practice

**Fig. 7:**
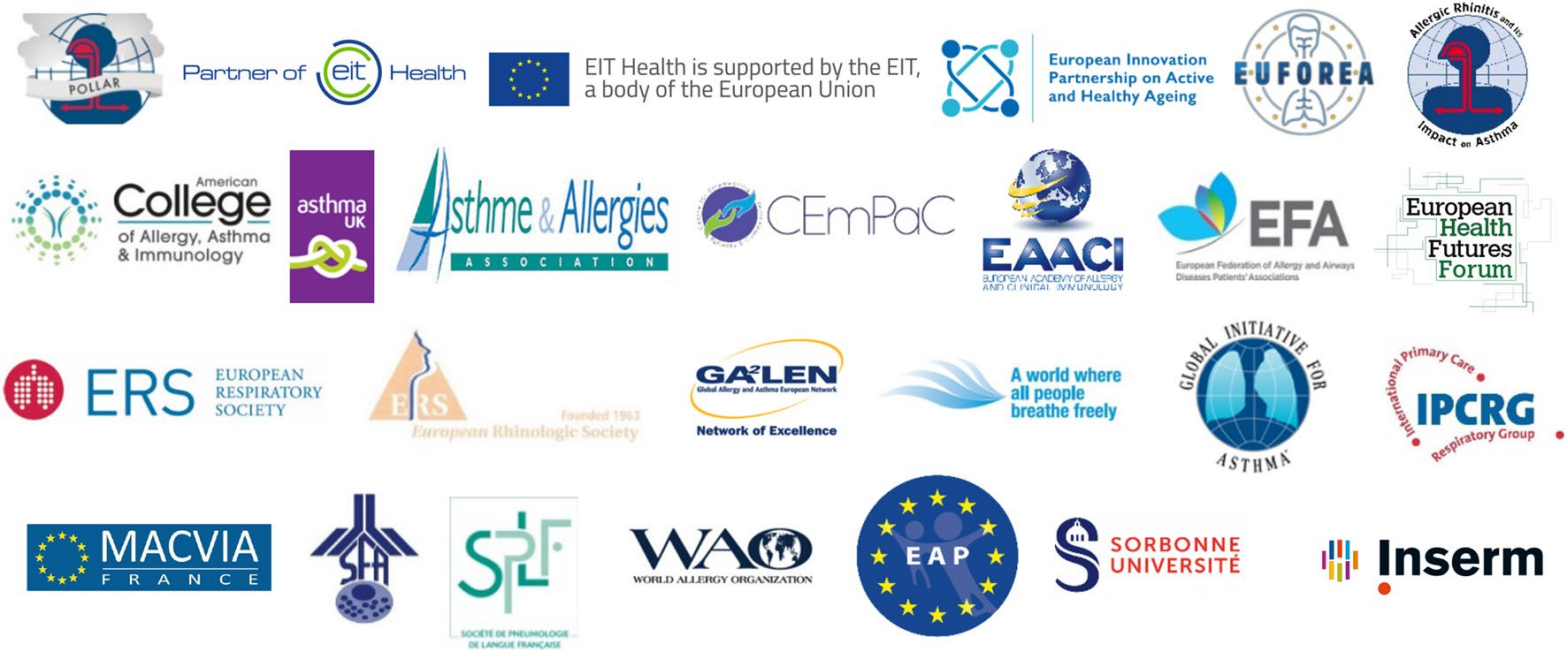
Sponsors of the meeting (Paris, December 3, 2018). POLLAR: Impact of Air POLLution in Asthma and Rhinitis, EIT Health: European Institute for Innovation and Technology, ARIA: Allergic Rhinitis and its Impact on Asthma, Euforea: European Forum for Research and Education in Allergy and Airways Diseases GA2LEN: Global Allergy and Asthma European Network, CEmPac: Centre for Empowering Patients and Communities, EAACI: European Academy of Allergy and Clinical Immunology, EFA: European Federation of Allergy and Airways Diseases Patients’ Associations, ERS: European Respiratory Society, ERS: European Rhinology Society, GARD: Global Alliance against Chronic Respiratory Diseases (WHO Alliance), GINA: Global Initiative for Asthma, MACVIA: Fondation VIA-LR, SPLF: Societé de Pneumologie de Langue Française, SFA: Société française d’Allergologie, WAO: World Allergy Organization

An expert meeting took place at the Pasteur Institute in Paris, December 3, 2018, to discuss next-generation care pathways and lessons learnt (Fig. 7, Annex 1): (1) patient participation, health literacy and self-care through technology-assisted “patient activation”, (2) implementation of care pathways by pharmacists and (3) next-generation guidelines assessing the recommendations of GRADE guidelines in rhinitis and asthma using real-world evidence (RWE) assessed by mobile technology. The meeting was organized by POLLAR and MASK in collaboration with GARD, patient’s organizations and all European scientific societies in the field.

## Abbreviations

AHA: active and healthy ageing
AIRWAYS ICPs: integrated care pathways for airway diseases
AR: allergic rhinitis
ARIA: allergic rhinitis and its impact on asthma
CDSS: clinical decision support system
CRD: chronic respiratory disease
DG CONNECT: directorate general for communications networks, content and technology
DG Santé: directorate general for health and food safety
EIP on AHA: European innovation partnership on AHA
EIP: European innovation partnership
EQ-5D: euroquol
Euforea: European forum for research and education in allergy and airways diseases
GARD: global alliance against chronic respiratory diseases
GP: good practice
HCP: health care professional
ICP: integrated care pathway
JA-CHRODIS: joint action on chronic diseases and promoting healthy ageing across the life cycle
MACVIA-LR: contre les MAladies chroniques pour un VIeillissement Actif (Fighting chronic diseases for AHA)
MASK: Mobile airways sentinel networK
MeDALL: Mechanisms of the development of ALLergy (FP7)
mHealth: mobile health
NCD: non-communicable disease
POLLAR: impact of air POLLution on Asthma and Rhinitis
QOL: quality of life
TRL: technology readiness level
VAS: visual analogue scale
WHO: World Health Organization
WPAI-AS: Work Productivity and Activity questionnaire
